# Supplementary material for: Identification and Functional Analysis of a Novel CTNNB1 Mutation in Pediatric Medulloblastoma
Source: Cancers (Basel). 2022 Jan 14;14(2):421. doi: 10.3390/cancers14020421 (PMC8773623; doi:10.3390/cancers14020421)
Supplement: Supplementary file 1 [file cancers-14-00421-s001.zip › Supplementary Figures S1,S2.pdf]

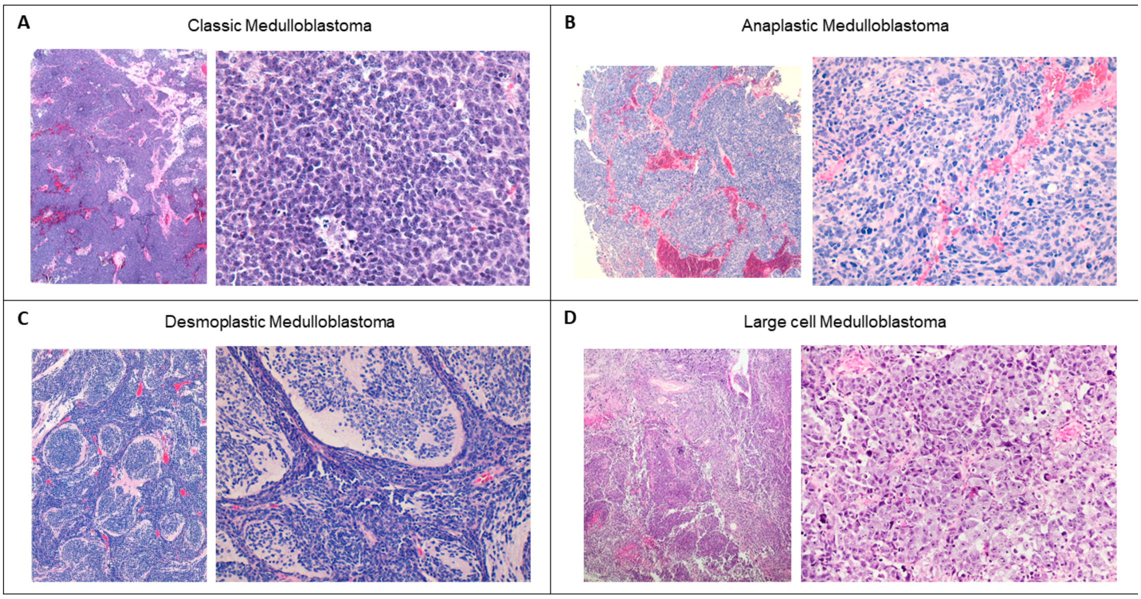

Supplementary Figure S1: Histologically defined entities of our medulloblastoma (MB) cohort. **Fig S1A:** Classic MB (Hematoxylin-eosin stain, left:40x magnification, righ:400x magnification). **Fig S1B**: Anaplastic MB (Hematoxylin-eosin stain, left:100x magnification, righ:400x magnification). **Fig S1C:** Desmoplastic MB (Hematoxylin-eosin stain, left:100x magnification, righ:200x magnification). **Fig S1D:** Large Cell MB (Hematoxylin-eosin stain, left:40x magnification, righ:400x magnification).

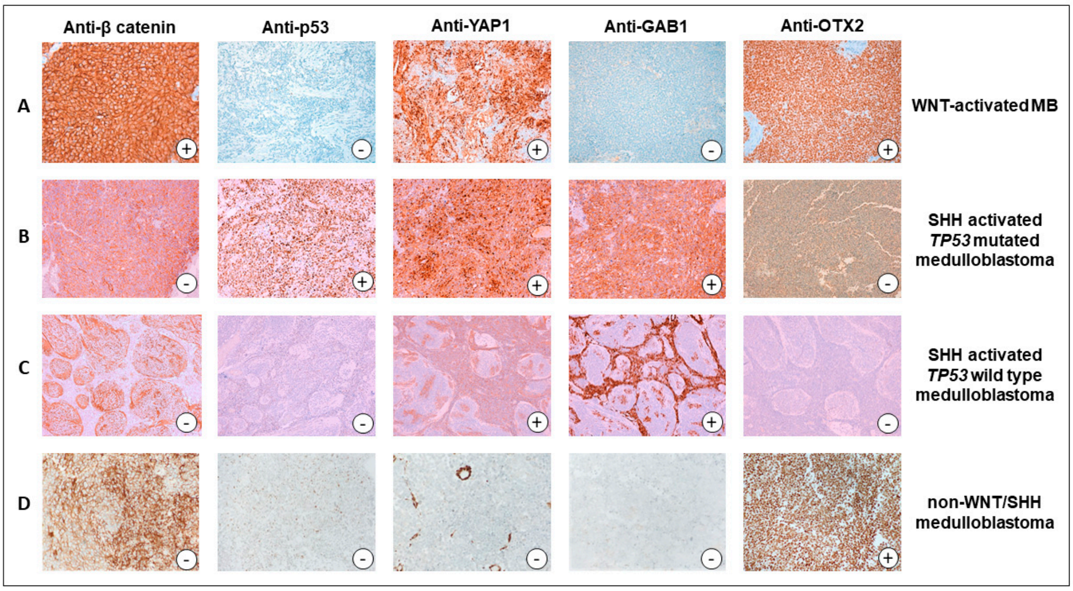

Supplementary Figure S2: Characteristic immunophenotype of the different molecular subgroups in our medulloblastoma (MB) cohort. **Fig S2A**: WNT-activated MB (200x magnification). **Fig S2B**: SHH-activated TP53 mutated MB (200x magnification). **Fig S2C**: SHH-activated TP53 wild type MB (100x magnification). **Fig S2D:** Non-WNT/SHH MB (200x magnification).
